# Blood biomarkers in early bacterial infection and sepsis diagnostics in feverish young children

**DOI:** 10.7150/ijms.69859

**Published:** 2022-04-11

**Authors:** Algirdas Dagys, Goda Laucaitytė, Augusta Volkevičiūtė, Silvijus Abramavičius, Rimantas Kėvalas, Astra Vitkauskienė, Lina Jankauskaitė

**Affiliations:** 1 Lithuanian University of Health Sciences, Medical Academy, Department of Pediatrics, 50161 Kaunas, Lithuania.; 2 Lithuanian University of Health Sciences, Medical Academy, Department of Laboratory Medicine, 50161 Kaunas, Lithuania.; 3 Laboratory of Preclinical Drug Investigation, Institute of Cardiology, Lithuanian University of Health Sciences, 50161 Kaunas, Lithuania.

**Keywords:** blood biomarkers, pediatric emergency department, sepsis, bacterial infection, cytokine, chemokine

## Abstract

Background and objectives: While most feverish children have self-limiting diseases, 5-10% develop a serious and potentially life-threatening bacterial infection (BI). Due to potential risk, prompt recognition of BI and sepsis in the pediatric emergency department (PED) remains a clinical priority. The aim of the study was to evaluate the role of certain cytokines and chemokines separately and in combination with routine blood tests in early BI and sepsis diagnostics at PED. Materials and methods: We prospectively studied children younger than 5 presenting to the PED with fever lasting for under 12 hours with high risk for serious illness. Clinical data, routine blood analysis, and inflammatory cytokine and chemokine panels were evaluated for their diagnostic abilities. Two separate analyses were carried out on the patients' data: one contrasting BI and viral infection (VI) groups, the other comparing septic and non-septic patients. Results: The sample comprised 70 patients (40% with BI). IL-2 was found to be the most specific biomarker to identify BI with specificity of 100%. The best discriminative ability was demonstrated by combining IL-2, IL-6, CRP, WBC, and neutrophil count: AUC 0.942 (95% Cl 0.859-0.984). IL-10 exhibited a greater AUC (0.837. 95% CI: 0.730-0.915 p<0.05) than CRP (0.807. 95% CI: 0.695-0.895 p<0.05) when predicting sepsis and showed high specificity (98%) and moderate sensitivity (75%). Conclusions: IL-6 and IL-2 could increase the diagnostic ability of routine blood tests for predicting BI, as IL-10 raises specificity for recognizing sepsis in the early hours of disease onset.

## Introduction

Fever is one of the most frequent conditions for young children to be referred to the pediatric emergency department (PED) 1-3. While most feverish children have self-limiting diseases, 5-10% develop a serious and potentially life-threatening bacterial infection (BI) like bacterial pneumonia, pyelonephritis, meningitis, or sepsis 2. Usually, early signs and symptoms of BI are subtle and clinically indistinguishable from a viral infection (VI), causing inappropriate patient management as well as antibiotic misuse 4,5. Consequently, all of the aforementioned factors can lead to unnecessary treatment or a prolonged disease state and treatment periods, resulting in medical complications and resistant pathogen development 5-7.

Introduction of new vaccines since 1990 has contributed to reduced incidence of infections caused by such dangerous pathogens as Haemophilus influenzae type b (Hib), Streptococcus pneumoniae, and Neisseria meningitidis 8-11. As a result, the mortality rate among children aged 5-14 years has halved since then 12. Unfortunately, infections continue to be the main cause of death in children under 5 years of age 12. According to the World Health Organization (WHO) report, sepsis ranks highly as a leading cause of death in the pediatric population not only in developing nations but also in developed countries 13.

Prompt recognition of BI and serious bacterial infection (SBI) in PED remains a clinical priority 4,7,14. The diagnostic value of vital signs is not sufficient in distinguishing between VI and BI 2,15. For example, hyperpyrexia (temperature over 40°C) is associated with increased risk for SBI only in infants under 3 months 16. Owing to the fact that a thorough clinical evaluation by the treating physician on its own is not sensitive enough 17,18, clinical assessment is usually supplemented with rapid laboratory tests. C reactive protein (CRP), complete blood count (CBC), and procalcitonin (PCT) are the most widely used diagnostic biomarkers in clinical practice, despite their limitations in sensitivity and specificity 19,20. In addition, blood cultures have been used as a gold standard for diagnosing bacteremia for many years, although it takes at least 24 hours to identify a pathogen microbiologically 21,22. Moreover, dozens of clinical decision models combining clinical signs and laboratory tests have been proposed for better detection of serious illnesses 23-26. Nevertheless, the majority of the current prediction rules have performed poorly in PED 26,27.

In this study, we aimed to evaluate the role of certain cytokines and chemokines separately and in combination with different routine blood tests in early bacterial infection and sepsis diagnostics in feverish children under the age of 5 admitted to PED.

## Materials and Methods

### Study design and patient characteristics

**Figure 1:**
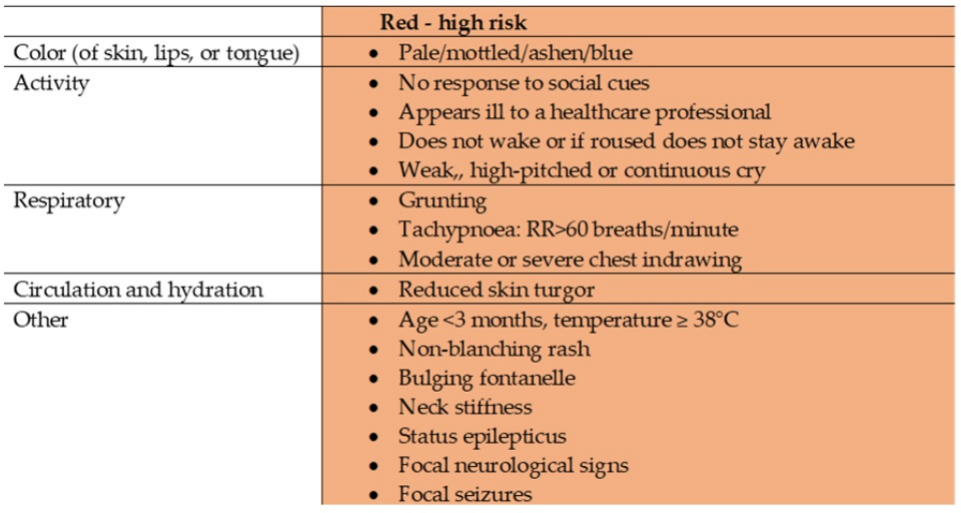
** Traffic light system for identifying risk of serious illness, NICE.** Adapted from “Assessment and initial management of feverish illness in children younger than 5 years: summary of updated NICE guidance” by E. Fields, J. Chard, S. M. Murphy and M. Richardson, 2013, BMJ, 346:f2866. Copyright 2013 by BMJ 28.

This was a prospective single-centre observational study conducted in the PED of the Lithuanian University of Health Sciences Kauno Klinikos (LUHS KK) Hospital between 01 January 2018 and 01 January 2019. All children under 5 years of age with a febrile illness lasting under 12 hours and with any of the symptoms or signs in the red column of the traffic light system following NICE guidelines “Fever in under 5s“ 28 (Figure 1) were included in the study. The exclusion criteria were as follows: neonates, antibacterial therapy prior to presentation to PED, organ transplantation, chronic infections, immunodeficiency, chronic inflammatory diseases, chromosomal anomalies, oncological diseases, psychiatric disorders, trauma, abuse, or parents' refusal to give their consent.

Permission to conduct this study was issued by Kaunas Regional Biomedical Research Ethics Committee (Protocol No.: BE-2-54, date: 2017-08-14) and the study was conducted in accordance with the Declaration of Helsinki and good clinical practice guidelines. Written informed consent was obtained from both parents of each patient.

### Data collection

All the patients included in the study were assessed by PED physicians according to the hospital's standard of care. Demographic data (age, gender) and vital signs (temperature, respiratory rate (RR), heart rate (HR), blood pressure (BP)) as well as laboratory tests (CBC, CRP) were recorded. Other necessary laboratory (urine test, lumbar puncture, blood, urine or stool culture) and instrumental (chest x-ray, ultrasound) diagnostic tests were done based on suspected illness and according to a local SOP (Standard Operating Procedure) for a feverish child. Additionally, blood was drawn into a Vacusera 2ml K3E/K3EDTA blood collection tube (Turkey (LOT 3365.0026.18)) for protein analysis. Samples were centrifuged according to our hospital's laboratory protocol and frozen at -20 °C within one hour of collection, pending further analysis.

Clinical diagnosis was made by a PED physician (emergency physician or pediatrician) after a careful examination and an evaluation of primary laboratory and instrumental tests. Thereafter, the patient was either discharged for outpatient care, monitored <24 hours in PED short-stay wards or hospitalised. All patients were followed up with until the final diagnosis was confirmed. The follow-up was achieved using multiple methods. Data collected electronically from hospital databases included test results, duration of admission and procedures during admission, the medical condition of the child, treatment, and the final diagnosis. Data were also collected during parental phone calls and included questions related to the disease of the child, namely, subsequent symptoms and additional diagnoses, if any, at another healthcare facility. Data of all subjects were evaluated by two independent investigators. In cases of differing opinions on the diagnosis, they were discussed separately with a group of three researchers. There were two separate analyses carried out on the patients' data: one contrasting BI and VI groups, the other comparing septic and non-septic patients.

The BI group included all patients with bacterial and severe bacterial infections, i.e. pneumonia (radiographically confirmed), meningitis (positive cerebrospinal fluid culture), sepsis (according to the International Pediatric Sepsis Consensus Conference on Pediatric Sepsis definitions for sepsis 30), bacterial enteritis/colitis (positive stool culture for pathogenic bacteria), pyelonephritis (pyuria (microscopic examination of urine sediment ≥10 leukocytes), significantly positive urine culture (>105 colony-forming units (CFU/mL)). BIs like tonsillitis, adenoiditis, otitis media, and scarlet fever were confirmed according to typical clinical signs (e.g. a bulging eardrum, pus-filled spots on tonsils, a positive strep test). Patients with an apparent VI were included in the VI group, provided that BI-related tests were negative and the patients recovered without antibiotic treatment within 14 days.

As healthy controls, we included children under 5 visiting the outpatients' clinic without any signs of acute infection. The exclusion criteria were the same as for the study group (neonates, antibacterial therapy, organ transplantation, chronic infections, immunodeficiency, chronic inflammatory diseases, chromosomal anomalies, oncological diseases, psychiatric disorders, trauma, abuse, or parents' refusal to give their consent). Children from the control group had their blood drawn for various reasons (e.g. preoperative screening before elective surgery, pre-school routine tests, vitamin D level analysis, etc.) with an extra blood sample taken for the same cytokine and chemokine panels as for the study subjects.

### Protein analysis

All patient's plasma samples were assessed for the same inflammatory cytokine and chemokine panels, such as interleukin-2 (IL-2), interleukin-6 (IL-6), interleukin-1β (IL-1β), interleukin-10 (IL-10), interleukin-12 (IL-12), interferon-γ (IFN-γ), interferon-β (IFN-β), triggering receptor expressed on myeloid cells 1 (TREM-1), and Lipocalin-2 (LCN-2)). All proteins were analysed via a multiplex assay approach using Luminex 100 (Human Magnetic Luminex assay, R&D Systems). Additionally, inducible nitric oxide synthase (iNOS) was measured by Human iNOS quantitative ELISA kit (Elabscience E-EL-H0753 ELIS) following the manufacturer's instructions. All assays were performed in duplicates and the averages were used for analysis.

### Statistical data analysis

Statistical analysis was performed by means of IBM SPSS 22 (SPSS, IBM Company, Armonk, New York, USA). The p-value of the Shapiro-Wilk test was used to test the data for normality. The data were not normally distributed so descriptive statistics are shown in median values with interquartile range (IQR). The nonparametric Mann-Whitney U test was used to perform a comparison between the two groups (p < 0.05 was considered to be statistically significant). Prognostic factors were analysed using the multivariate logistic regression analysis. Multivariate logistic regression was conducted to calculate the coefficients of biomarker combinations when used in predicting BI and sepsis. Cytokine concentrations required a log transformation to satisfy the linearity assumption. The variables yielding *p*-values below 0.2 by univariate analysis were entered into a forward multivariate logistic regression analysis. The multivariate analysis results were summarised by estimating odds ratios and the relevant 95% confidence intervals (Cls). A receiver-operating characteristic (ROC) curve was developed for biomarkers and their combinations, presenting the area under the curve (AUC) with a 95% confidence interval (CI). The Youden's index was used to determine cut-off values. Following the cut-off values, sensitivity and specificity were obtained by assessing and comparing differential diagnostic abilities of individual biomarkers and biomarker combinations in discriminating between BI and VI as well as between sepsis and non-sepsis groups.

## Results

### Patient characteristics

A total of 70 pediatric patients with a mean age of 21 months (IQR: 10-31 m) who matched inclusion criteria were enrolled; 53% were males. The average duration of fever (at the time of arrival to PED) was 7 hours (IQR: 3-10 h) (Table 1). Out of all recruited patients, 60% (n=42) were diagnosed with VI, 40% (n=28) had BI, sepsis developed in 6% patients (4). Final diagnoses of the patients are listed in Table 2.

### Comparison of serum inflammatory markers between bacterial and viral infection patients

**Figure 2:**
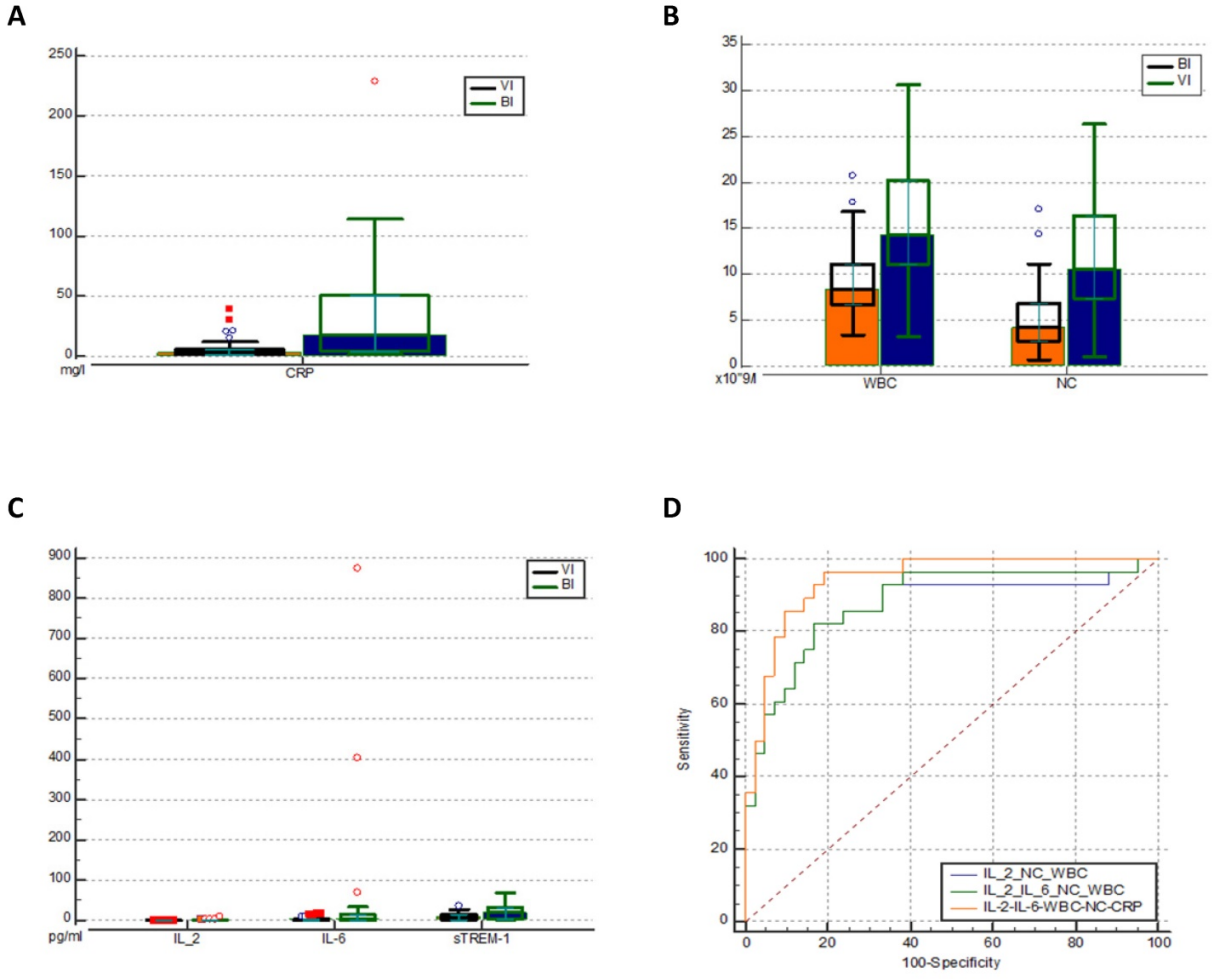
Comparison of CRP (mg/l) levels (**a**) WBC and NC (pg/ml) levels (**b**) and Il-2, IL-6 and sTREM-1 levels (**c**) in the two groups. ROC analysis of different combinations of biomarkers for predicting BI (**d**). IL - interleukin; CRP - C reactive protein; WBC - white blood cells; NC - neutrophil count. VI -viral infection, BI - bacterial infection, sTREM-1- soluble triggering receptor expressed on myelocytes 1, ROC - receiver operating characteristic curve.

As expected, in patients with BIs CRP levels (Figure 2a), leukocytes (WBC), and neutrophil count (NC) (Figure 2b) were significantly higher than in those with VIs (Table 3). Also, we observed statistically significant increases of IL-2, IL-6, and sTREM-1 levels in the BI group (Figure 2c). IL-6 (cut-off value: 2 pg/ml) was shown to be the most sensitive biomarker when distinguishing bacterial from viral infections with sensitivity of 68% and specificity of 69%. In contrast, IL-2 (0.3 pg/ml) was found to be the most specific biomarker with specificity of 100% and sensitivity of 28%. However, it demonstrated a low AUC of 0.610 (0.486-0.724) which indicates poor predictive accuracy. As for sTREM-1, we found low sensitivity and moderate specificity (54% and 86% respectively). In addition, none of them outperformed the discrimination capacity of CRP, WBC or NC.

In order to maximize sensitivity and specificity, we analysed different combinations of several blood biomarkers. The results showed that including IL-2 increased the overall performance of WBC (10 × 10^9^/l) and NC (7.5 × 10^9^/l) as a combination of biomarkers with a pooled AUC 0.867 (95% Cl 0.770-0.965) achieving 100% of sensitivity and 67% of specificity. However, the best discriminative ability was demonstrated by combining IL-2, IL-6, CRP, WBC, and NC: AUC 0.942 (95% Cl 0.859-0.984) (Figure 2d), showing sensitivity of 96% and specificity of 81% (Table 4). The cut-off values, AUC, sensitivity, specificity of different biomarkers and their combinations are described in Table 4 (extended table with positive and negative predictive values can be found in Supplementary Table S3).

### Comparison of serum inflammatory markers between sepsis and non-sepsis patients

**Figure 3:**
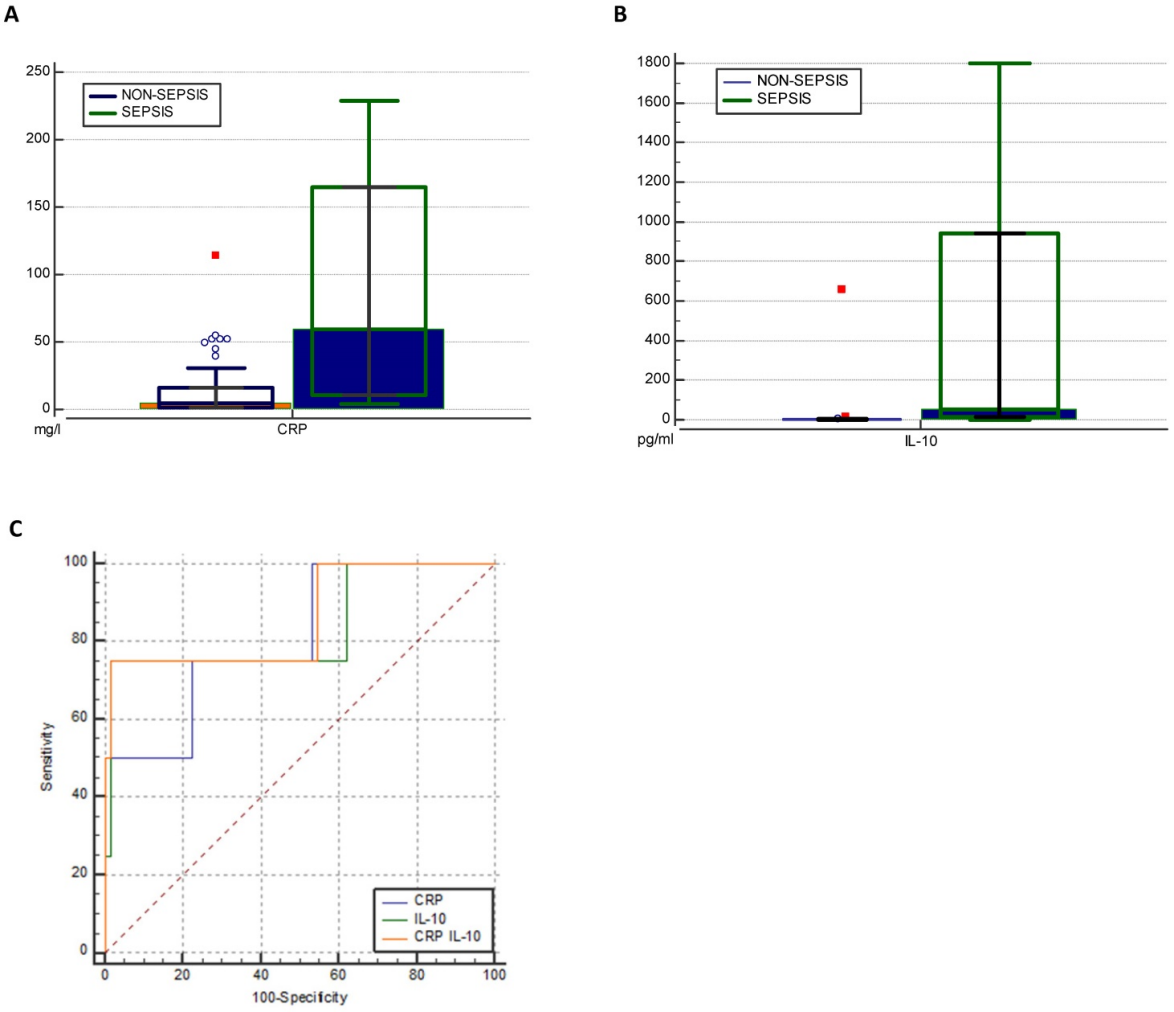
Distribution of levels of CRP (mg/l) (**a**) and IL-10 (pg/ml) (**b**) in two groups. ROC analysis of CRP, IL-10 and combination of both biomarkers in predicting sepsis (**c**). IL, interleukin; CRP, C reactive protein; WBC, white blood cells; NC, neutrophil count.

Higher levels of IL-6 (202.92 to 1.81 pg/ml) and IFN-γ (1.74 to 0.28 pg/ml) were found in the sepsis compared to the non-sepsis patients' group, although differences were not statistically significant. Correspondingly, septic patients had double the levels of sTREM-1 (24.60 to 10.79) as well as LCN-2 (42506.90 to 26861.55). However, non-septic children had lower levels of IL-1β (1.80 for sepsis, 2.22 for non-sepsis pg/ml), but the difference was not significant (Table 3). Septic patients had statistically significantly higher levels of IL-10 (54.40pg/ml to 2.38 pg/ml) (Figure 3b). At the cut-off value of 14 pg/mL, IL-10 exhibited a greater AUC (0.837, 95% CI: 0.730-0.915, p<0.05) than CRP (0.807, 95% CI: 0.695-0.891, p<0.05) and showed high specificity (97%) with moderate sensitivity (75%) (Table 4). To increase overall performance for discriminating sepsis we combined CRP with IL-10 and found a greater AUC of 0.860 (Cl 95% 0.756-0.931) (Figure 2c), while increasing specificity (98%) but not sensitivity (75%).

Multivariate logistic regression was conducted to calculate the coefficients of the biomarkers' combinations when used in predicting BI and sepsis (Supplementary Table S1 and Table S2).

Levels of all cytokines and chemokines, except IL-12, were statistically significantly lower in healthy controls than in the study groups. In addition, levels of IL-2 in the VI group were the same as in the healthy controls.

## Discussion

Early BI diagnostics and a timely initiation of antibiotic therapy improve prognosis and treatment outcomes, which can be life-saving for children 4. Despite a variety of novel biomarkers, discriminating bacterial from viral infections as well as early detection of sepsis remain great challenges in PED. The purpose of our research was to investigate whether the use of inflammatory cytokines and chemokines adds any diagnostic value for detecting bacterial infection and sepsis in the emergency department.

When comparing the BI and VI groups, we found statistically significant differences in levels of IL-2, IL-6, and sTREM-1. In contrast, the levels of almost all the cytokines and chemokines were elevated in the sepsis group compared to the non-sepsis group but it was only the levels of IL-10 that yielded a statistically significant difference.

Although the results of a systematic review and meta-analysis conducted by Jiyong et al. have shown that the sTREM-1 test could be used as a helpful diagnostic criterion for BI 31, we found low sensitivity and moderate specificity for sTREM-1 (54% and 86% respectively) in distinguishing BI from VI. As for sepsis diagnostics, previous studies have indicated the potential value of sTREM-1 in evaluating disease severity and outcome in septic adults 32-34 and neonates 35-37. However, only a few studies have been done with septic children older than 1 month and those demonstrated moderate diagnostic potential of this biomarker at best 38.

IL-6 is typically defined as an acute phase pro-inflammatory cytokine, although it can also have anti-inflammatory effects 39,40. It has been documented that IL-6 becomes elevated in blood earlier than CRP, i.e. during the course of BI its concentration increases within the first 6 hours 41. Unsurprisingly, IL-6 is hence a widely studied biomarker in the pediatric population. Lacour et al. analyzed 133 children and found elevated levels of IL-6 in the SBI group, as a result being able to predict the SBI diagnosis in children with fever without source 40. Moreover, Heikkinen et al. demonstrated the discriminatory power of IL-6 for pneumococcal acute otitis media (AOM) 42. Another study carried out in 2018 found an association between increased levels of IL-6 and pneumococcal infection among children under 5 years of age, hospitalized with community-acquired pneumonia (CAP) 43. IL-6 has been most extensively studied within neonatal populations and is generally regarded as a sensitive marker of acute bacterial infection 44. Additionally, it has superior sensitivity to CRP in neonatal sepsis diagnostics 45-48. According to a meta-analysis by Sun et al., 48 31 studies with a total of 3076 neonates showed that the specificity and sensitivity of IL‐6 for the diagnosis of neonatal sepsis were 88% (95% CI: 83%‐92%) and 82% (95% CI: 77%‐86%) respectively. In our study, only 2 out of 4 septic patients had high IL-6 values (874.46 and 403.44 pg/ml). In the other two, IL-6 values were low (0.46 and 2.41 pg/ml). However, this difference could have been a result of the different arrival times (even if early, i.e. <12 hours after initial fever) leading to differences in the blood collection times. Unfortunately, we cannot perform an analysis of this hypothesis due to the small sample size. Although it is true that the septic patients had higher IL-6 levels as compared to the non-sepsis group, the difference was not statistically significant. Another hypothesis could be individual variations in the specifics of the host-response and severity of sepsis. Further investigation with a larger sample size and fixed times for blood collection is needed.

According to Biron et al., IL-10 is the key cytokine in anti-inflammatory response and its increase is significantly associated with sepsis as well as worse outcomes, including death 41. To add more, IL-10 demonstrated good results in excluding bacteremia and clinical sepsis in oncology patients with febrile neutropenia 49. Other research studies found that IL-6 values decreased rapidly over 12 hours, while IL-10 levels tended to increase faster and persist for longer, which makes it a valuable biomarker in sepsis diagnostics 50. The performance of IL-10 in our study was roughly consistent with previous research and indicated a large AUC, high specificity of 98%, and moderate sensitivity of 75%.

As many researchers have previously indicated 21, we also found that combinations of blood biomarkers yield additional diagnostic value over individual tests. In addition, in the early hours of disease onset routine inflammatory markers are not elevated enough to suspect serious illness 51. In such cases, other biomarkers may be crucially important. In our research, CRP, WBC, and NC had extremely low cut-off values in the early hours of infection. This means that clinicians would not suspect BI or sepsis, relying only on regular inflammatory markers. We demonstrated that a combination of WBC, NC, CRP, IL-2, and IL-6 offered very high sensitivity of 96%, specificity of 81%, and a large AUC 0.942 (Cl 95% 0.859-0.984) in predicting BI, exceeding the individual performance of any of the studied biomarkers alone. Similarly, combining CRP with IL-10 had high discriminative ability with AUC 0.860 (Cl 95% 0.756-0.931) and increased specificity (from 77% to 98%) but not sensitivity (75%) in detecting septic patients.

The present study had some limitations, the most significant being the small sample size. Due to that, patients' data were not divided into different age groups and we were not able to compare BI and SBI groups separately. Another important factor which could have influenced the study was the lack of rapid viral testing in PED. Moreover, larger-scale proteomic study could be performed with regard to identifying some novel proteins as early markers of SBI and/or sepsis. Concerning the application of those biomarkers or their combinations in practice, further investigation is needed, including research on older children and children in risk groups, such as cancer patients or presenting with other chronic conditions.

## Conclusions

Our study was supplemented with additional information about the role of cytokines and chemokines in early diagnostics of BI and sepsis in young children in PED. According to our results, when used in addition to routine blood tests, IL-6 and IL-2 can improve overall diagnostic ability in predicting BI, while IL-10 could increase specificity in early sepsis recognition. Although these early results are promising, they require additional empirical validation due to the small study size. In sum, further investigation of the relevant biomarkers, especially in combination with routine inflammatory marker analysis, has the potential to improve PED diagnostic practices considerably.

## Supplementary Material

Supplementary tables.

## Figures and Tables

**Table 1:** Patient characteristics

Data characteristics	Study group N=70	Control group N= 16
Male, N	37	9
Female, N	33	7
Age, months, median (Q1-Q3)	21 (10-31)	19 (11-33)
Time of fever, h, median (Q1-Q3)	7 (3-10)	-

**Table 2:** Final diagnoses

Infection	No.
**Viral infections**	***N (42)***
Upper respiratory tract infection	32
Gastroenteritis	7
Bronchiolitis	1
Enteroviral infection	1
Other	1
**Bacterial infections**	***N (28)***
Tonsillitis	5
Adenoiditis	2
Otitis media	2
Scarlet fever	1
Pneumonia	6
Sepsis	4
Bacterial enteritis or colitis	4
Pyelonephritis	2
Meningitis	1
Unspecified	1

**Table 3:**
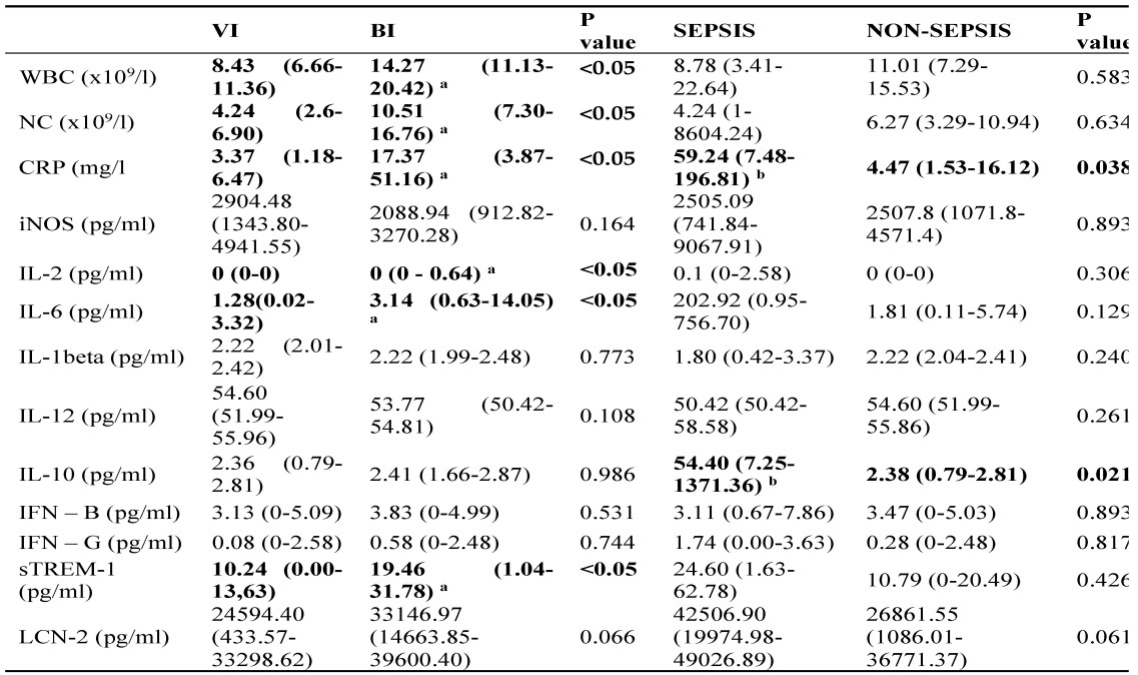
The serum biomarkers medians (IQR) in patients of different groups

**Abbreviations:** WBC, leukocytes; NC, neutrophil count; CRP, C reactive protein; iNOS, inducible nitric oxide synthase; IL, interleukin; IFN, interferon; sTREM-1, soluble triggering receptor expressed on myeloid cells 1; LCN-2, Lipocalin-2; BI, bacterial infection; VI, viral infection; P value < 0.05 rejects the null hypothesis that the distributions of both groups are identical; ^a^ P<0.05 compared to viral infection; ^b^ P<0.05 compared to non-sepsis; Data are shown in median values with interquartile range.

**Table 4:**
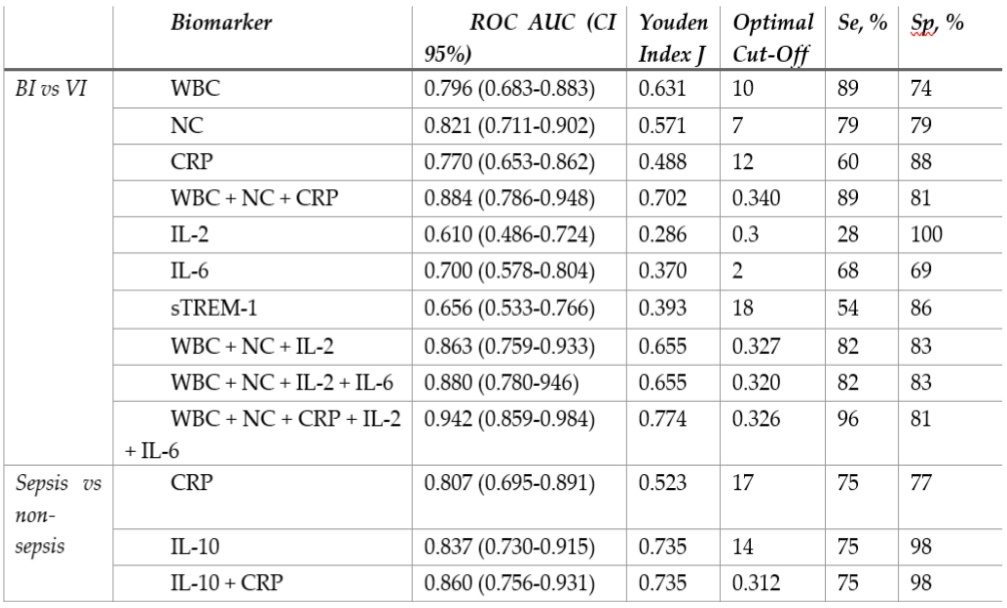
Receiver-operating characteristic curve analysis and diagnostic performance of different biomarkers and their combinations

BI, bacterial infection; VI, viral infection; WBC, leukocytes; NC, neutrophil count; CRP, C reactive protein; IL, interleukin; sTREM-1, soluble triggering receptor expressed on myeloid cells 1; AUC, area under curve; Se, sensitivity; Sp, specificity.

## Abbreviations

AUC: area under the curve
BI: bacterial infection
BP: blood pressure
CBC: complete blood count
CI: confidence interval
CRP: C-reactive protein
Hib: Haemophilus influenza b
HR: heart rate
IFN-β: interferon-β
IFN-γ: interferon-γ
IL-10: interleukin-10
IL-12: interleukin-12
IL-1β: interleukin-1β
IL-2: interleukin-2
IL-6: interleukin-6
iNOS: inducible nitric oxide synthase
IQR: interquartile range
LCN-2: lipocalin-2
PCT: procalcitonin
PED: pediatric emergency department
ROC: receiver-operating characteristic
RR: respiratory rate
SBI: serious bacterial infection
sTREM-1: soluble triggering receptor expressed on myeloid cells 1
VI: viral infection
WHO: world health organization
