# Supplementary material for: Blood biomarkers in early bacterial infection and sepsis diagnostics in feverish young children
Source: Int J Med Sci. 2022 Apr 11;19(4):753–61. doi: 10.7150/ijms.69859 (PMC9108404; doi:10.7150/ijms.69859)
Supplement: Supplementary file 1 — Supplementary tables. [file ijmsv19p0753s1.pdf]

Supplementary material

Table S1. Univariate and multivariate logistic regression models to predict bacterial infection

		**Univariate logistic regression**					95% CI	
		Coeff. B	S.E.	Wald	p	Odds ratio	Lower	Upper
1	WBC	0.21	0.06	13.55	<0.05	1.24	1.11	1.39
	constant	-3.02	0.76	15.87	<0.05	0.05		
2	NC	0.27	0.07	14.97	<0.05	1.31	1.14	1.50
	Constant	-2,50	0.60	17.48	<0.05	0.08		
3	CRP	0.08	0.02	10.35	<0.05	1.08	1.03	1.13
	Constant	-1.41	0.37	14.28	<0.05	0.24		
4	IL-2	3.01	1.85	2.64	0.1	20.31	0.54	768.93
	Constant	-0.76	0.28	7.32	<0.05	0.47		
5	IL-6	0.09	0.04	3.83	<0.05	1.09	1.00	1.19
	Constant	-0.89	0.32	7.73	<0.05	0.41		
		**Multivariate logistic regression**					95% CI	
		Coeff. B	S.E.	Wald	p	Odds ratio	Lower	Upper
1	NC	0.14	0.17	0.67	0.41	1.15	0.83	1.59
	CRP	0.06	0.03	4.29	0.04	1.06	1.00	1.12
	WBC	0.13	0.16	0.75	0.39	1.14	0.84	1.55
	IL-2	2.20	1.69	1.70	0.19	9.00	0.33	244.94
	IL-6	0.05	0.06	0.71	0.40	1.05	0.93	1.19
	Constant	-4.50	1.25	13.05	0.00	0.01		
2	WBC	0.09	0.12	0.52	0.47	1.09	0.86	1.39
	NC	0.19	0.14	1.82	0.18	1.21	0.92	1.59
	IL-2	2.44	1.92	1.61	0.20	11.45	0.26	496.90
	Constant	-3.35	0.91	13.47	0	0.04		
3	WBC	0.065	0.136	0.227	0.634	1.067	0.817	1.393
	NC	0.191	0.149	1.637	0.201	1.210	0.904	1.620
	CRP	0.062	0.026	5.695	0.017	1.064	1.011	1.119
	Constant	-3.483	1.002	12.077	0.001	0.031		
4	NC	0.136	0.166	0.666	0.414	1.145	0.827	1.586
	CRP	0.057	0.028	4.288	0.038	1.059	1.003	1.118
	WBC	0.135	0.155	0.752	0.386	1.144	0.844	1.551
	IL-2	2.197	1.686	1.699	0.192	9.001	0.331	244.936
	IL-6	0.053	0.063	0.713	0.398	1.055	0.932	1.194
	Constant	-4.503	1.247	13.047	0.000	0.011		

WBC – leukocytes; NC – neutrophil count; CRP – C reactive protein; iNOS – inducible nitric oxide synthase; IL – interleukin; S.E. – standard error; C.I. – confidence intervals

Table S2. Univariate and multivariate logistic regression models to predict sepsis

	**Univariate logistic regression**	95% CI

		Coeff. B	S.E.	Wald	p	Odds ratio	Lower	Upper
1	CRP	0.031	0.014	4.913	0.027	1.032	1.004	1.061
	constant	-3.841	0.818	22.064	0	0.021		
2	IL-10	0.003	0.002	2.566	0.109	1.003	0.999	1.008
	Constant	-3.175	0.610	27.044	0	0.042		
		**Multivariate logistic regression**					95% CI	
		Coeff. B	S.E.	Wald	p	Odds ratio	Lower	Upper
1	CRP	0.04	0.02	4.93	0.03	1.04	1.00	1.07
	IL-10	0	0	2.18	0.14	1.00	1.00	1.01
	Constant	-4.67	1.15	16.52	0	0.01		

CRP – C reactive protein; IL – interleukin; S.E. – standard error; C.I. – confidence intervals

Table S3. Extended Table 4 (receiver-operating characteristic curve analysis and diagnostic performance of different biomarkers and their combinations) with positive and negative predictive values.

	Biomarker	*ROC AUC (CI 95%)*	*Youden Index J*	*Optimal Cut-Off*	*Se, %*	*Sp, %*	*PPV %*	*NPV %*
BI vs VI	WBC	0.796 (0.683-0.883)	0.631	10	89	74	69	91
	NC	0.821 (0.711-0.902)	0.571	7	79	79	71	85
	CRP	0.770 (0.653-0.862)	0.488	12	60	88	77	77
	WBC + NC + CRP	0.884 (0.786-0.948)	0.702	0.340	89	81	64	88
	IL-2	0.610 (0.486-0.724)	0.286	0.3	28	100	100	68
	IL-6	0.700 (0.578-0.804)	0.370	2	68	69	60	76
	sTREM-1	0.656 (0.533-0.766)	0.393	18	54	86	71	74
	WBC + NC + IL-2	0.863 (0.759-0.933)	0.655	0.327	82	83	68	90
	WBC + NC + IL-2 + IL-6	0.880 (0.780-946)	0.655	0.320	82	83	71	93
	WBC + NC + CRP + IL-2 + IL-6	0.942 (0.859-0.984)	0.774	0.326	96	81	75	95
Sepsis vs non-sepsis	CRP	0.807 (0.695-0.891)	0.523	17	75	77	16	98
	IL-10	0.837 (0.730-0.915)	0.735	14	75	98	75	98
	IL-10 + CRP	0.860 (0.756-0.931)	0.735	0.312	75	98	50	98

BI – bacterial infection; VI – viral infection; WBC – leukocytes; NC – neutrophil count; CRP – C reactive protein; IL – interleukin; sTREM-1 - soluble triggering receptor expressed on myeloid cells 1; AUC – area under curve; Se – sensitivity; Sp – specificity; PPV – positive predictive value; NPV – negative predictive value
